# Foraging dispersion of Ryukyu flying-foxes and relationships with fig abundance in East-Asian subtropical island forests

**DOI:** 10.1186/s12898-017-0146-8

**Published:** 2017-11-14

**Authors:** Ya-Fu Lee, Yen-Min Kuo, Hsin-Yi Chang, Chi-Feng Tsai, Shigeyuki Baba

**Affiliations:** 1 Department of Life Sciences, National Cheng Kung University, Tainan, 701 Taiwan; 2 Tropical Biosphere Research Center and Faculty of Agriculture, University of the Ryukyus, Okinawa, Japan; 3 Present Address: International Society for Mangrove Ecosystems, University of the Ryukyus, Okinawa, Japan

**Keywords:** Abundance, Bats, Chiroptera, Figs, Flying-foxes, Frugivores, Iriomote island

## Abstract

**Background:**

Figs are widely distributed key resources to many tropical-subtropical animals, and flying-foxes are major consumers and seed dispersers of figs. Bat-fig interrelationships, however, may vary among species differing in fruiting traits, i.e., bat- versus bird-dispersed figs. We examined Ryukyu flying-fox foraging dispersion and the relationships with tree species composition and fig abundance in forests of Iriomote Island.

**Results:**

Bat foraging dispersion showed no spatial patterns with respect to different areas of the island, and was not explained by heterogeneity, density, or basal area (BA) of total trees, nor by relative density or BA of fruiting trees or total fruiting figs among sites. Instead, bat densities were positively dependent on the relative density of total figs, and particularly the relative BA of bat-dispersed figs *Ficus septica* and *F. variegata*. Both species were dominant figs in forests, fruiting asynchronously with long crop seasons, and were used as predominant foods. Bats foraged mostly solitarily and the mean density was in a hump-shaped relationship with crop sizes of the dominant bat-figs. These two species and *Ficus benguetensis* are larger-sized bat-figs, all contained more seeds, higher dry-pulp mass and water mass, but not necessarily water content. By approximate estimation, higher proportions of seeds of these bat-figs would have been removed from fruits through the bat consumption, than that of small-sized bird-figs like *F. virgata, F*. *superba*, and *F*. *microcarpa*.

**Conclusions:**

The foraging dispersion of Ryukyu flying-foxes in forests depends on the availability of the most abundant bat-figs that serve as predominant foods. Intermediate levels of crop sizes of theses figs appear most fit with their solitary foraging. Our results suggest that as density and BA coverage of these dominant bat-figs are below a certain level, their effectiveness to attract bats may dwindle and so would their chance of dispersal by bats.

## Background

The *genus Ficus* (Moraceae) is widely distributed in tropical areas with its distribution and diversification centering around the equatorial Indomalay-Australasian region and extending poleward to around 35°N and 35°S [1, 2]. The 800 or so extant *Ficus* species constitute one of the most speciose genera of flowering plants. They occur in diverse habitats in various growth forms and engage with their pollinators (i.e., agaonid wasps) in complex and intriguing coevolutionary relationships [3–8].

*Ficus* plants play a key ecosystem role in contributing to the diversity of global tropical forests [9, 10]. The majority of known *Ficus* species produce edible inflorescences (syconia), so-called figs, that are largely apparent, accessible, easy to handle by various volant, arboreal, or even terrestrial animals, and nutritionally attractive [11, 12]. Many *Ficus* species have multiple crops year round, with asynchronous ripening phases among plants or even within a plant, which enhances their availability to foragers [9, 13]. This can be particularly crucial in lean times when other plants cease to produce fruits (but see [14, 15]). Globally, a variety of frugivorous or omnivorous vertebrates, predominantly birds and mammals that comprise nearly 1300 species, have been identified as fig-eating to various extents [12], although probably few depend solely on figs [13].

Among mammalian families, the Old World fruit bats (pteropodids) account for the highest proportion (ca. 16.5%) of the 284 species of fig-eating mammals, with at least 47 species confirmed [12]. They appear all rely on figs as predominant food resources [12, 16–19]. In return, pteropodid fruit bats are significant seed dispersers of *Ficus* and various other plants, including many large-seeded fruits, in the Indomalayan and Australasian regions [20–23]. Yet, dominant palaeotropical plants in early successions are rarely bat-dispersed except the Moraceae, which is comprised mostly of *Ficus* [24]. This further pinpoints potential effects of fruit bat-fig interactions on forest structure, successional patterns, and fate of large-seeded fruits, particularly in areas where suitable seed-dispersers may be scarce, such as remote oceanic islands [20].

*Ficus* plants may be categorized based on certain noticeable traits of fig morphology and phenology and their associated major dispersers [25]. Unlike bird-dispersed figs, those that are preferentially fed on by phyllostomid fruit bats in the Neotropics (i.e., bat syndrome figs, hereafter as bat-figs) are generally green or dull in color, aromatic, span a wider range in fig size among species, and ripen more synchronously [25, 26]. Greater variation occurs with species that are typified as bird syndrome figs (i.e., small-sized and orange-red in color, hereafter as bird-figs) and may be fed on by mixed forager groups, particularly in the Old World tropics [27, 28]. On the other hand, pteropodids appear attracted to functionally dioecious (gyno-dioecious) *Ficus* species [27] that are more diverse and abundant than monoecious species in the Indomalay-Australasian region. Dioecious figs that fail to attract birds are predicted to produce smaller crops of larger-sized and dull-colored figs [12].

We examined foraging dispersion patterns of pteropodid bats and their relationships with fig assemblages in the subtropical forests of an Indomalayan oceanic island. The dispersion of foraging pteropodids may be affected by factors associated with land use patterns, habitats, and food availability (e.g., grey-headed flying fox *Pteropus poliocephalus* in Australia [29]). We tested the hypothesis that the availability of bat-figs affects nocturnal foraging dispersion of pteropodids and predicted that bat presence and abundance will depend on the availability of bat-figs that serve as predominant food resources. Frugivorous bats and birds are often attracted to fleshy fruits that are characterized by high water content [3, 30]. When consuming figs, however, pteropodid bats typically compress fruits against the ridged palate with the tongue, so the pulp-juice is squeezed out and swallowed along with numerous tiny seeds [12, 31]. Bats then eject deformed pulps as pellets, instead of swallowing the whole fig as do most birds [12, 17, 31]. Thus different water content in figs may contribute to different proportions of seeds being ingested, which indirectly affect the chance of seeds being further dispersed [32, 33]. We additionally tested that proportions of seeds remaining in ejected pellets are affected by water content, and predicted that figs containing higher water content aid to higher proportions of seeds being removed from fruits through the feeding process of fruit bats.

## Methods

### Study area and species

Iriomote (24°15′ ~ 25′N, 123°40′ ~ 55′E) is located at the southernmost tip of the Ryukyu Islands. It is the largest among the nine islets of the Yaeyama Islands, with an area of 289 km^2^ and its highest peak at 470 m above sea level. This subtropical island is typified by hot and humid summers and warm but windy winters. Temperatures generally rise above 25 °C from May to October, peaking at ca. 29 °C in July, and descend to 17 ~ 18 °C in January, with a mean annual rainfall of over 2300 mm (Japan Meteorological Agency data).

Intact primary or secondary broadleaf forests cover about 80% of this mountainous inland and are characterized by *Castanopsis* spp., *Quercus* spp., *Schima* spp. and various figs (*Ficus* spp.). *Hernanadia nymphaeifolia* (Presl) Kubitzki and horsetail pine *Casuarina equisetifolia* L. are common in coastal forests, whereas *Bruguiera gymnorrhiza* (L.) Lam, *Rhizophora mucronata*, and *R*. *stylosa* mangroves prevail along estuaries and rivers. *Ficus*, such as cedar fig *F*. *superba* Miq. and white fig *F*. *virgata* Blume, are mixed with *Cerbera manghas* L., *Heritiera littoralis*, hanging-flower checkerboard foot *Barringtonia racemosa* (L.) Blume *ex* DC, and screwpine *Pandanus odoratissimus* L. f. in swampy wetland forests [34]. Human residences and cultivated areas are located in lowlands below 100 m along the shoreline and are more prominent on the eastern and northern coasts.

The near-threatened Ryukyu flying-fox (*Pteropus dasymallus* Temminck, 1825) is among the most northerly distributed pteropodids [35, 36]. Its five subspecies are each narrowly distributed but collectively cover a broad latitudinal range along the West Pacific island chain from Tokara Islands, Okinawa Island, Yaeyama Islands, and small islets of the Nansei Islands in southern Japan to Batan and other northern Philippine islets [35, 37]. Among the subspecies, *P. d. yayeyamae* occurs on most of the Yaeyama Islands [35]. In Iriomote, *F. septica* Burm. f. and *F*. *variegata* Blume are their predominant foods, followed by *F*. *benguetensis* Merr., banyan (*F. microcarpa* L. f.), and at least 35 other fig and non-fig species [17].

### Forest structure and tree composition

We conducted field surveys adopting the point-centered quarter method [38, 39] to assess forest structure in July, 2012, along 12 forest transects 1-km in length each. Four transects were established on each of the west, north, and east sides of the island. These transect sites had been previously surveyed for flying-fox abundance and activity [17], making comparisons between the two assessments possible. We did not survey the southern-most side of the island where forest transects were limited by rough terrain, and the southwestern-most corner, Funauki, due to its inaccessibility by land.

In each transect site, we randomly picked six 100-m long sections and six random points within each section to collect measurements using 2-digit random numbers from 01 to 99 in increasing order. We specifically kept the difference of any two successive numbers greater than five, so each pair of random points selected were at least 5 m apart and individual trees would not be measured repeatedly [40, 41]. Our sampling proceeded from the coast toward the forest interior, and the smallest random number selected was set as the initial point along a transect line. At each sampling point, we determined four quarters divided by the transect line and its perpendicular line. In each quarter, the nearest tree to the sampling point with at least 4 cm in diameter was located. The quarter, distance from the nearest tree to the sampling point, species, and CCH (circumference at chest height of 130 cm [42]) were recorded, and fruiting or blooming trees were noted. We calculated density and basal area (BA) using distance and CCH data [41] for total trees, total fig trees, fruiting trees, and fruiting fig trees at each site, and obtained the relative density and relative BA of total fig trees, fruiting trees, and fruiting fig trees in relation to that of total trees [41]. We calculated the relative frequency of occurrence (FO) and relative abundance (RA) for each tree species at each forest site and for the entire sampling across sites. We further adopted and modified Curtis and McIntosh’s [43] use of the arithmetic mean of these standardized measures to obtain an estimate of the relative importance (RI) of each species in samples [17]. The converted Simpson index, 1 − *D* = 1 − Σ (*p*_*i*_^2^ *)*, was used to assess the heterogeneity (SH) in species composition [39], where *p*_*i*_ is the relative abundance of particular species *i* (*i* = 1 to *s*, *s* being the total number of species in a sample).

### Foraging bat dispersion and abundance

From 28 June to 22 September, 2012, we conducted 50 bat surveys along the 12 transect sites (mean 4.2 ± 0.11 nights per site) where forest tree composition was assessed. Transect lines followed previously adopted outskirt routes leading toward inland forests [17], except that Aira-gawa and Nanama-gawa forest sites replaced two routes where no traces of bats were recorded. Any two proximate transects were roughly 2 ~ 4 km apart, and collectively they covered areas ranging up to 5 km from the coast. In each survey run, we alternated among the western, northern, and eastern sites of the island and randomly picked transects until all transects were assessed over a period of 2 ~ 3 weeks. We also alternated the nightly proceeding direction within any transect so that no point was ever visited at the same time.

We arrived at a site at least 30 min before sunset to observe bat arrivals or passing until sunset. Assessments began within 30 min after sunset and ended usually within 2 h, and transects were walked at roughly 1 km/h. A group of 2 ~ 3 workers searched for bats with binoculars (Leica 10 × 42 BN, Solms, Germany), aided with head- or spotlights and by bat sounds while feeding or interacting with each other. Upon each encounter, we tallied the number of bats present and recorded the species of trees where bat perched or searched for fruits, perch heights, and bats’ behaviors (e.g., moving, searching, feeding, individual interactions). We restricted our searches to a strip of 30 m on either side and assumed a complete census. Our prior tests indicated that this is a suitable distance in most habitats, but in inner forests we acknowledge that observations were more limited. After each transect survey, we remained following and monitoring bats present until past the midnight or an hour since the last bat observed left a site, whichever came first.

### Fig phenology, fruit sampling, and feeding traces

Along with each bat survey at each transect, we assessed tree phenology in the early afternoon, sampled bat feeding traces after each bat survey, and sampled fresh mature fruits at dawn. We estimated crop size in the mature edible stages (i.e. the post-floral phase defined in [44]) of each fruiting monoecious fig tree and for dioecious female figs that produced seed-carrying syconia. We visually estimated crop sizes for trees with few or small amounts of fruits [45], but applied a stratified sampling method [26] when fruits were too many to count reliably. For each tree, we divided branches into three classes and defined the largest fig-bearing branches as the 3rd class, which merged into the 2nd class and then further merged into the 1st class, often bifurcating directly from the main trunk. We randomly selected 30 tertiary branches, tallied the number of figs on each branch, and then obtained the mean value (*n*). The number of the 1st class branches was counted (*b*_*1*_), then six 1st class branches were randomly selected to estimate the mean number of 2nd class branches per 1st class branch (*b*_*2*_) and the mean number of the 3rd class branches per 2nd class branch (*b*_*3*_). Total crop size was estimated as crop size = *n* × *b*_*1*_ × *b*_*2*_ × *b*_*3*_ [25].

We randomly collected accessible mature figs (the stage E [44]) directly from each fig tree where bat feeding was observed. Ryukyu flying-foxes usually drop ejecta pellets beneath the feeding tree, only occasionally mouth-carrying a large fruit to a feeding perch [46 YFL unpubl. data], thus we searched feeding traces underneath each feeding fig tree after our nightly observations, mostly discarded pellets but also culled fruits and fecal samples. Fresh mass (fm) and volume size of intact fruits and pellets were measured, and the numbers of seed they contained were tallied. We obtained dry mass (dm) of intact fruits and pellets after oven drying overnight at 50 °C. Water mass (wm, g) was determined as the difference between fresh fruit mass and dry fruit mass and water content (%) was calculated as WC = wm/fm × 100 [47]. It was not possible to count the actual number of seeds of each fig fed on by bats, particularly in field conditions. Thus we used the difference in mean seed numbers between sampled fresh intact fruits and ejecta pellets to obtain an approximate estimate of the proportions of seeds being removed away from different species of fig fruits after bat feeding.

### Data analyses

Data are presented as the mean ± standard error (SE) unless otherwise noted. Statistical tests were conducted using Statistica 12.0 (StatSoft, Tulsa, USA) with the significance level set at *α* = 0.05. Proportional data were arcsine-transformed to meet the normality requirement [48]. We assessed the correlation (Pearson’s *r*) between relative frequency of occurrence (FO) and relative abundance (RA) for dominant tree species, among tree density, basal area (BA) coverage, and heterogeneity for forest transect sites, and between bat densities estimated over sites between the 2005 and 2012 assessments. A χ^2^ test was used to determine if the frequency distribution was random among abundance levels. We used analysis of variance (ANOVA) to examine the effects of site location on variances in bat density. Multivariate analysis of variance (MANOVA) was also used to examine the effects of site location on variances in tree density, BA coverage, and heterogeneity, and that in mean volume size, seed number, dry-pulp mass, water mass, and water content of fresh fruits and pellets among different fig species. We used additional multiple-range comparisons (HSD for unequal sample sizes) to locate the differences when significant differences were observed. We performed multiple regression analysis to examine the relationship of bat abundance with density, BA coverage, and heterogeneity of total trees among forest sites. The same analysis was conducted with relative density and relative BA for fruiting trees, total fig trees, fruiting fig trees, and selected predominant food fig species (i.e., *F. septica* and *F. variegata* combined), respectively. Linear regression was also conducted to examine the respective relationships of bat density and pellet number with canopy volume [48].

## Results

### Tree composition and forest structure

**Fig. 1:**
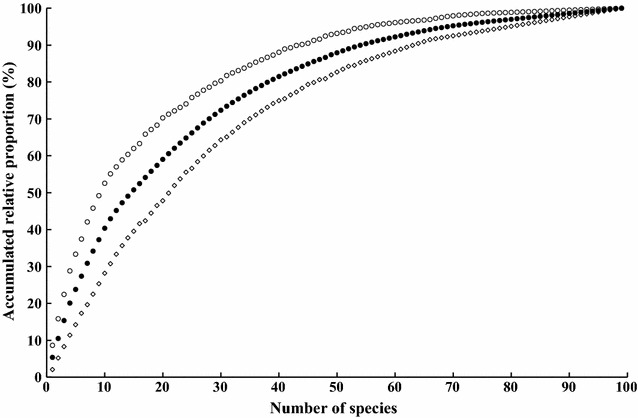
Accumulated proportions of relative abundance (○; RA), relative frequency of occurrence (□; FO), and relative importance (●; RI) values of tree species as number of species identified increased at the forest sites assessed in Iriomote Island, Japan. Curtis and McIntosh’s [43] use of the arithmetic mean of RA and FO was adopted to obtain an estimate of the relative importance

We sampled 1725 trees in 103 species of 51 families, including 10 species of figs. Less than a quarter (22 species) of the species, however, collectively accounted for 72% of relative abundance (RA), nearly 52% of relative frequency of occurrence (FO), and over 62% of relative importance (RI; Fig. 1). These 22 trees were widely distributed and more abundant than the rest of the species across sites (FO-RA correlation: *r* = 0.79, *p* < 0.05). Exceptions included harlequin glory-bower (*Clerodendrum trichotomum* Thunb.) and Malaysian persimmon (*Diospyros maritima* Blume), which were not substantially abundant, whereas white cedar (*Melia azedarach* L.) and Chinese guger-tree (*Schima superba* Gardner & Champ.) were locally abundant in restricted sites (Table 1).

**Table 1:** The dominant tree species and their respective relative frequency of occurrence (FO), abundance (RA), and importance values (RI)

Species (Family)	FO	RA	RI
*Bischofia javanica* (Euphorbiaceae)*	1.81^20^	1.61^17^	1.71^15^
*Clerodendrum trichotomum* (Verbenaceae)	2.07^13^	0.89	1.48^22^
*Diospyros maritima* (Ebenaceae)*	2.07^13^	1.01	1.54^21^
*Elaeocarpus sylvestris* (Elaeocarpaceae)*	2.07^13^	1.19^22^	1.63^19^
*Ficus benguetensis* (Moraceae)*	2.07^13^	1.31^20^	1.69^16^
*F. septica* (Moraceae)*	2.84^5^	3.33^10^	3.09^10^
*F. variegata* (Moraceae)*	3.10^1^	6.37^4^	4.73^4^
*F. virgate* (Moraceae)*	2.07^13^	1.25^21^	1.66^18^
*Fraxinus formosana* (Oleaceae)	2.84^5^	3.39^9^	3.12^9^
*Leucaena leucocephala* (Mimosaceae)	2.84^5^	3.75^8^	3.30^8^
*Macaranga tanarius* (Euphorbiaceae)*	3.10^1^	6.60^3^	4.85^3^
*Mallotus japonicus* (Euphorbiaceae)	3.10^1^	4.11^7^	3.60^6^
*Melanolepis multiglandulosa* (Euphorbiaceae)*	2.84^5^	4.52^6^	3.68^5^
*Melia azedarach* (Meliaceae)*	1.29	1.96^13^	1.63^19^
*Pinus luchuensis* (Pinaceae)	2.07^13^	8.63^1^	5.35^1^
*Rhus succedanea* (Anacardiaceae)*	3.10^1^	7.20^2^	5.15^2^
*Schefflera octophylla* (Araliaceae)*	2.58^9^	1.90^14^	2.24^12^
*Schima superba* (Theaceae)*	0.78	2.56^11^	1.67^17^
*Styrax japonica* (Styracaceae)	2.58^9^	2.56^11^	2.57^11^
*Trema oreintalis* (Ulmaceae)*	2.33^11^	1.90^14^	2.12^13^
*Trochodendron aralioides* (Trochodendraceae)	2.33^11^	4.64^5^	3.48^7^
*Turpinia ternata* (Staphyleaceae)*	2.07^13^	1.49^18^	1.78^14^

Superscript values indicate respective ranking

* Food plants of Ryukyu flying-foxes [17, 53]

Moraceae and Euphorbiaceae each contributed four dominant tree species, accounting for a combined 33.2% importance, and were represented by 11 and 9 species, respectively. Wax tree (*Rhus succedanea* Linn.), large-leaved tree (*Macaranga tanarius* (L.) Műll.Arg.), *F*. *variegata*, *Melanolepis multiglandulosa* [(Reinw. ex Blume) Rchb.f. & Zoll.], and *F. septica* topped others in both measures, collectively contributing a total importance value of 21.5%, and all are fed on by Ryukyu flying foxes (Table 1). *Ficus benguentensis* and white fig ranked relatively low among these top 22 species, whereas the rest of the figs, including dioecious *F. ampelas*, *F. erecta*, and *F. irisana*, and monoecious *F. caulocarpa*, *F. microcarpa*, and *F*. *superba*, altogether accounted for only 2.88% of the total importance value.

On average, each transect-site contained 34.8 ± 1.6 species of trees (range: 21 ~ 41). Among sites, however, tree density, basal area coverage (BA), and heterogeneity fluctuated, and we found no apparent spatial patterns (Mann–Whitney *U*-test, all *p* values > 0.05) in any of the three variables compared among the western, northern, and eastern island sites (Table 2). Tree densities (*r* = − 0.21) and basal areas (*r* = − 0.12) each was not correlated to tree heterogeneity, nor to one another (*r* = 0.29; all *p* values > 0.05).

**Table 2:** Mean (± SE) linear density of fruit bats (bats/km-h) of the 12 forest transect sites and their respective tree heterogeneity, density (trees/ha), and basal area coverage (BA; m^2^/ha)

Transect site^§^	Bat density	Heterogeneity	Tree density	BA
^w^Shirahama	5.53 ± 1.14^3^	20.47^3^	334.23	10.003
^w^Midara	10.28 ± 2.49^2^	16.01	376.19	29.274^3^
^w^Sonai	2.94 ± 0.69	3.22	777.15^2^	15.962
^w^Hoshidate-Inab	1.42 ± 0.44	18.62	637.45^5^	17.860
^n^Uehara	3.0 ± 0.11^6^	18.01	645.26^4^	13.745
^n^Funaura	1.52 ± 0.20	16.14	527.11	32.821^2^
^n^URE	2.42 ± 0.98	14.99	600.85^6^	19.931^5^
^n^Nishida-gawa	5.22 ± 1.29^4^	19.76^4^	675.73^3^	27.403^4^
^e^Aira-gawa	1.54 ± 0.40	19.43^5^	943.12^1^	33.830^1^
^e^Komi	3.49 ± 1.29^5^	28.21^1^	584.00	19.573^6^
^e^Otomi	2.64 ± 1.50	19.14^6^	462.44	11.140
^e^Nakama-gawa	12.33 ± 2.16^1^	20.93^2^	519.60	18.328

Superscripts indicate the top six sites with the highest ranking in each measurement

^§^w: west, n: north, e: east

### Bat abundance and relationships with forest structure and tree composition

**Fig. 2:**
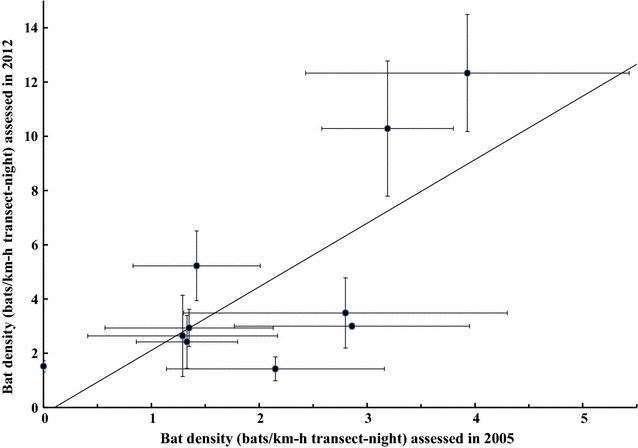
Mean (± SE) foraging bat density (bats/km-h transect-night) assessed in this study and its correlation with that assessed in 2005 at the same forest sites in Iriomote Island, Japan, based on the same protocols

We recorded 416 fruit bats in 357 sight encounters (1.2 ± 0.03 bats per encounter; range: 1 ~ 5) over a total survey time of 2691.9 min in 50 transect-nights (53.67 ± 2.09 min per transect-night). On average, we recorded 7.1 ± 4.99 bat encounters (range 0 ~ 34) with an overall encounter probability of 98% across the entire assessment, and sighted 8.3 ± 5.19 bats (range 0 ~ 40) per transect-night. Incorporating the transect lengths and survey time, our data translated to a mean density of 4.4 ± 1.02 bats/km-hr transect-night, with a variance/mean ratio of 2.85. We sighted singles and paired bats in 88.8 and 7.3% of encounters, respectively, and bats in groups of three or larger in only 3.9% of cases (*χ* ^2^ = 1061.42, d.f. = 4, *p* < 0.001). The highest abundances occurred in the Nakama-gawa and Midara forest transects, followed by the Shirahama and Nishida-gama transects, whereas the lowest occurred in Aira-gawa, Funaura, and Hoshidate-Inab. Overall mean density did not differ among the western (5.04 ± 1.94), northern (3.04 ± 0.79), and eastern sites (5.0 ± 2.48, *F*_(2, 9)_ = 0.37, *p* = 0.699; Table 2). Estimated bat abundances, however, were positively correlated to previous assessments at the same sites using the same protocol in 2005 (*r* = 0.73, *p* < 0.05; Fig. 2).

**Fig. 3:**
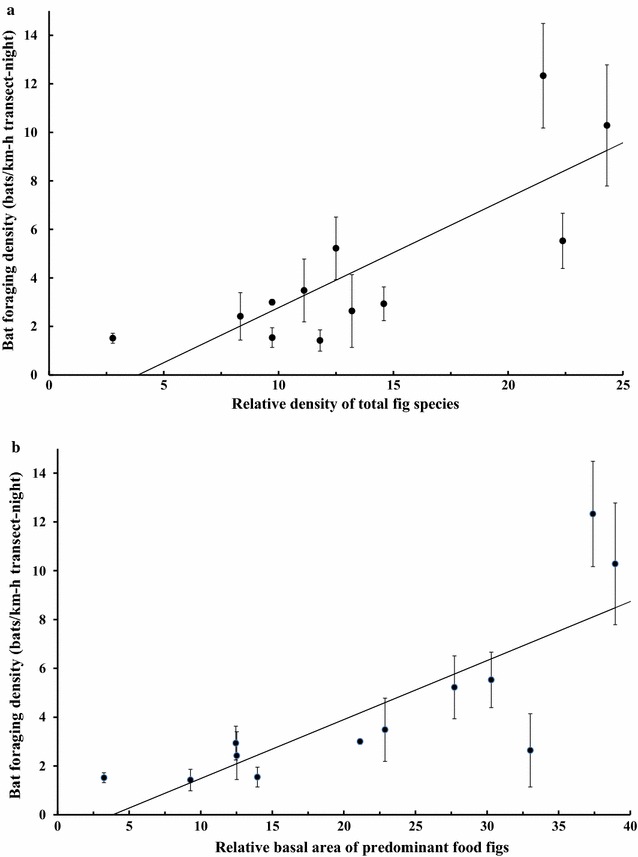
The relationships of mean (± SE) foraging bat density with (**a**) the relative density of total figs and (**b**) the relative basal area coverage of predominant food plants *Ficus variegata* and *Ficus septica* combined at the forest sites in Iriomote Island, Japan. The relative density and relative basal area of any subsamples of forest trees were calculated as in relation to that of total trees [41]

Foraging bat density was not explained by the heterogeneity, density (DS), or basal area (BA) of total trees (*r* ^2^ = 0.257, *F*_(3, 8)_ = 0.921, *p* = 0.474) among forest sites. Examining the finer subsets of forest composition, including fruiting trees, total figs, fruiting figs, and figs as predominant food resources of bats (i.e., *F*. *septica* and *F*. *variegata*), we found no dependence of bat abundance on relative density or BA coverage of total fruiting trees (*r* ^2^ = 0.174, *F*_(2, 9)_ = 0.944, *p* = 0.591) or fruiting figs either (*r* ^2^ = 0.103, *F*_(2, 9)_ = 1.633, *p* = 0.248). Yet bat abundance was positively dependent on relative density of total figs (*r* ^2^ = 0.628, *F*_(2, 9)_ = 10.954, *p* < 0.005; *y* = 0.814 *DS* 1.754, *p* < 0.05; Fig. 3a) and relative BA coverage of predominant figs (*r* ^2^ = 0.609, *F*_(2, 9)_ = 18.113, *p* < 0.005; *y* = 0.242 *BA* 0.933, *p* < 0.05; Fig. 3b).

### Fruiting figs, fig consumption, and seed removal

**Fig. 4:**
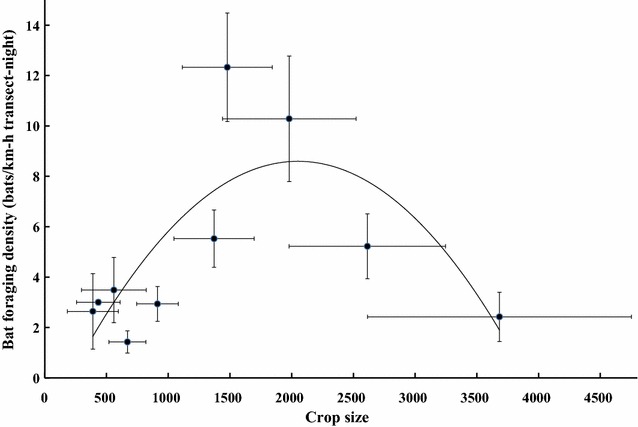
A hump-shaped relationship of mean (± SE) foraging bat density with crop sizes of *Ficus variegata* and *Ficus septica* combined, which were the most dominant bat-figs in forests and served as predominant food plants to Ryukyu flying-foxes in Iriomote Island, Japan [17]

We recorded the fruiting phenology of 114 trees in eight fig species, comprising 50 *F. variegata*, 27 *F. septica*, 14 *F*. *benguetensis*, and 10 *F. virgata*, which are dioecious and typically fruited over the entire study period but ripened asynchronously. Monoecious *F*. *cauloarpa*, *F*. *microcarpa*, and *F*. *superba* accounted for only about 9%, and the fruiting period of each fig tree lasted less than 3 weeks. Most pellets retrieved were *F. variegata* (44.3%) and *F. septica* (46.2%, *n* = 558; Table 3). Canopy volumes of fruiting figs were highly correlated to the numbers of pellets retrieved underneath (*r* = 0.894, *F*_(2, 9)_ = 44.99, *p* < 0.001), but only slightly positively related to bat abundances (*r* = 0.602, *F*_(2, 9)_ = 7.232, *p* < 0.05). In contrast, fruit bat abundance was in a hump-shaped relationship with the crop sizes of predominant food figs (*F*. *septica* and *F*. *variegata*; Fig. 4).

**Table 3:** Mean (± SE) fruit volume (mm^3^), dry mass (dm, g), water mass (wm, g), water content (wc, %), ripe color, and seed numbers in mature fresh fruits and ejecta pellets analyzed for monoecious^1^ vs. dioecious^2^ figs from three subgenera

Species		Volume	DM	WM	WC	Ripe color	Seed #	Seed prop.
Sycidium (Sect. Palaeomorphe)								
*F. virgata* ^2^	Fruit (2/17)	694.08 ± 43.50	0.25 ± 0.09	0.65 ± 0.11	0.75 ± 0.03	Dark purple	136.50 ± 9.32	
	Pellet (2/10)		0.18 ± 0.02	0.31 ± 0.12	0.59 ± 0.12		54.25 ± 9.04	39.7
Sycomorus (Sect. Sycocarpus)								
*F. bengue* ^2^	Fruit (3/15)	3445.35 ± 291.04**	1.02 ± 0.09**	3.08 ± 0.18**	0.75 ± 0.02	Dark green	1182.50 ± 65.97**	
	Pellet (3/14)		0.56 ± 0.07	1.1 ± 0.16	0.63 ± 0.05		183.40 ± 29.37	15.5
*F. septica* ^2^	Fruit (16/106)	5368.62 ± 213.26**	1.46 ± 0.06**	4.92 ± 0.17**	0.78 ± 0.01	Yellow-green	1129.39 ± 98.72**	
	Pellet (16/258)		0.48 ± 0.02	1.08 ± 0.05	0.60 ± 0.02		92.91 ± 6.05	8.2
Sycomorus (Sect. Neomorphe)								
*F. variegata* ^2^	Fruit (13/141)	8004.55 ± 281.16**	1.47 ± 0.05**	7.13 ± 0.23**	0.83 ± 0.003*	Yellow-green	1681.76 ± 49.58**	
	Pellet (13/247)		0.52 ± 0.02	1.69 ± 0.08	0.71 ± 0.01		93.42 ± 8.59	5.5
Urostigma (Sect. Urostigma/Sect. Conosycea)								
*F. superba* ^1^	Fruit (2/16)	786.58 ± 43.89	0.16 ± 0.02	1.06 ± 0.16	0.86 ± 0.02*	Red-purple	99.06 ± 8.37	
	Pellet (2/19)		0.11 ± 0.02	0.39 ± 0.05	0.74 ± 0.05		69.13 ± 10.39	69.8
*F. microcarpa* ^1^	Fruit (2/12)	849.86 ± 128.34	0.22 ± 0.01	0.54 ± 0.10	0.75 ± 0.03	Red-purple	34.92 ± 4.95	
	Pellet (2/10)		0.09 ± 0.01	0.15 ± 0.02	0.58 ± 0.04		–	–

Sample sizes (tree/fruit) are in parenthesis. Proportions of mean seed numbers remaining in pellets relative to that in fig fruits were estimated. Species associated with a value with asterisks indicates a significantly greater value for fresh mature fruit than that of other species without asterisks under the same variable

–, proportion was not estimated for *F. microcapra* because bats while feeding on *F*. *microcarpa* often culled multiple fruits in mouth, which resulted in pellets that might comprise remains of uncertain number of actual fig fruits

*p < 0.05, ** *p* < 0.01

Sycomorus figs (*F. variegata*, *F. septica*, and *F*. *benguetensis*) were significantly greater in fruit size, contained more seeds, and higher dry-pulp mass and water mass (Pillai’s trace *V* = 0.985, *F*_(20, 1052)_ = 17.19, *p* < 0.001) than urostigma figs (*F*. *superba* and *F*. *microcarpa*) and *F. virgata* (all *p* values < 0.01 in paired comparisons). Water content was higher in *F*. *superba* and *F. variegata* than in other species (*p* values < 0.05; Table 3). In approximate estimation, lower proportions of seeds remained in pellets of *F. variegata*, *F. septica*, and *F*. *benguetensis.* Seed proportion in each of these species being removed from fruits via bat feeding was about 1.7 ~ 1.4 times and 3.1 ~ 2.8 times that in *F. virgata* and *F. superba*, respectively.

## Discussion

A previous assessment over multiple habitats in Iriomote indicates higher bat abundances in areas with lower land proportions under cultivation and human residences, and higher tree heterogeneity and fruit-tree densities in villages [17]. Our present study instead focused solely on inland forests that serve as roosting and also main foraging habitats of fruit bats. We found no correlation of bat abundance with tree heterogeneity, nor to density and basal area of total trees. Tree heterogeneity, density, and basal area varied among forest sites across the island with no apparent spatial patterns, as did bat abundance. Yet, bat abundances were comparable and significantly positively correlated to those previous data assessed with the same methods in the same forests [17]. This seeming difference in dispersion patterns pinpoints the necessity for a finer examination of habitat and resource use by fruit bats in forested areas.

Our data indicate that the abundance of fruit bats foraging in forests positively depended on the relative density of fig trees, and the relative basal area of fruiting *F*. *variegata* and *F*. *septica*, but not that of total fruiting figs. Both species are characterized by the typical traits of bat-dispersed figs, including size, color (but note regional variation in ripe colors of *F*. *variegata* [49, 50]) and presumably scent (e.g., monoterpenes, [51]). *Ficus variegata* and *F*. *septica* were also the most common figs at our sites. This conforms to our prediction that fruit bat abundance depends on the availability of the most abundant bat syndrome figs, but not necessarily on bird-figs, and provides support for the dispersal syndrome hypothesis based on the color, size, and scent of figs [25, 50].

Phenologically, bat-dispersed figs are assumed to ripen more synchronously [26], yet dioecious bat-figs display complex patterns, being inter-tree asynchronous, or inter-tree synchronous but intra-tree asynchronous and with a relatively long crop season [49, 52, 53]. Being largely solitary, fruit bats in Iriomote find ample food supplies by spreading out while foraging where there are abundant bat-figs in forested areas. In contrast, monoecious figs are intra-tree synchronous with an apparent fruit availability peak and a shorter crop period. Bats feeding on these figs are likely forced to aggregate and potentially cause resource competition. This is consistent with our finding of higher mean bat abundances at intermediate crop sizes, as that noted in frugivorous birds [54]. We also observed aggressive behaviors like fighting screams, wing-beating display, and chasing each other, on occasions when multiple bats perched and fed on the same trees (YFL unpubl. data).

Both *F*. *septica* and *F. variegata* are the predominant foods of fruit bats in Iriomote [17]. This concurs with the relationship of bat abundance with fig tree abundance. In addition to external traits, *F*. *septica* and *F. variegata* contained higher dry pulp mass and may be nutritionally advantageous as well. For instance, *F*. *variegata* contains a high calcium content [55] that is essential for animals and fruit bats often obtain it by folivory [56]. *Ficus septica* and *F*. *variegata*, along with other palaeotropical dioecious figs (mostly of the sycomorus group), also tend to contain higher proportions of carbohydrates and sugars favored by frugivores than many monoecious figs assessed [55]. In contrast, monoecious figs like *F*. *caulocarpa* and *F*. *superba*, although higher in lipids and fiber, are lower in calcium and protein than dioecious sycidium figs (e.g., *F*. *irisana* and *F*. *ampela* [57]).

As food resources, *F*. *variegata* and *F*. *septica* were among the dominant trees in our forest sites. *Ficus benguetensis* (a bat-fig) and *F*. *virgata* (a bird-fig) ranked in the last quarter of the top 22 species, and all the rest of the bird-figs were ranked much lower in importance. On the other hand, *F. microcarpa and F. superba* have been widely planted as common ornamental trees in urban or human residential areas throughout their distributional range in tropical-subtropical East Asia [49, 53]. This may explain the observations that Ryukyu flying foxes elsewhere may feed on more diverse items in forests but use *F. microcarpa* as a core food plant in urbanized areas [18]. Urbanization is less intensive in Iriomote and fruit bats are still capable of finding a sufficient food supply in more natural habitats. Yet in places within or near villages with abundant *F. microcarpa*, bats were observed frequently visiting and feeding substantially at crop peaks (YFL, unpubl. data).

This is consistent with an opportunistic foraging mode adopted by flying-foxes with spatiotemporal variation that reflects local phenology, availability, and the diversity of fruits [17, 29, 31, 58, 59]. In addition, a mix of fig species often provides a more complete set of nutrients [60]. In contrast, major avian frugivores in Iriomote such as brown-eared bulbuls (*Hypsipetes amaurotis*), emerald doves (*Chalcophaps indica*), whistling green pigeons (*Treron formosae*), and white-eyes (*Zosterops japonica*) are all gape-limited and feed only on small-sized bird-figs like *F*. *microcarpa* and *F*. *superba* [46, 53, YFL unpubl. data]. Other pigeons or doves (e.g., *Columba, Streptopelia*) may even be seed predators [12, 21, 22]. Fruit bats in Iriomote thus likely play an extremely important role, and also represent a very low redundancy, in seed dispersal of those larger-sized figs [61].

Compared to bird-figs, mean remaining seeds in pellets of those large-sized bat-figs were much lower than that in fresh fruits. This suggests that higher proportions of seeds in large bat-figs may be more easily removed from fruits through bat feeding, and concurs with the tight inter-dependent relationship between fruit bats and bat-figs. Our estimates were less precise due to lack of direct fig-pellet match in seed counts, yet obtaining seed counts of fresh fruits prior to bat feeding is impossible or impractical in the field or feeding experiments. We collected fresh figs from parent trees where bat feeding and pellet ejection were observed, and pellets were collected timely and directly beneath parent trees. The chance and extent of secondary seed removal by other animals would be very slight or trivial. Given the observed variation, the dramatic difference in seed remains between bat- and bird-figs suggests further considering distinctive traits that separate them. Yet, our results don’t fully support the prediction regarding water content that is complicated by dry pulp mass and fruit size. Instead, water mass contained by bat-figs was significantly higher than that in bird-figs. Water mass in fruits generally rises with fruit size [62], whereas fig pulp mass tends to increase at a lowered rate depending on the investment on seeds [63]. Further studies may examine water mass of figs over a broader size range and the effect of water on seed removal from fruits through the feeding of bats by more detailed behavioral experiments.

Yet, the function and effectiveness of fruit bats in seed dispersal may cease as bat abundance drops below certain thresholds [64, 65], due to various factors. In Fiji, landscape mosaics and declining forest habitats have resulted in the preferential foraging in farmland by Pacific flying-foxes, *Pteropus tonganus* [66]. This seemingly suggests flexible and adaptive behavior by fruit bats; however, nutrient deficiencies may drive bats to feed on a diversity of plants, as cultivated plants have largely replaced native species, especially figs [67]. Habitat fragmentation and alteration unavoidably force fruit bats to travel over an even larger area for foods [68] or enter places where food can be found with relative ease (e.g., *Pteropus* in Australia [29]), yet threats to fruit bats facing a changing world will also increase, such as conflicts with human economic demands [69, 70]. This is evidenced by the recently revealed incidents of illegally killing fruit bats on Yaeyama Islands [71], that along with other factors has drawn alarming calls for reassessments and reconsideration of its status [36].

## Conclusion

Foraging dispersion of Ryukyu flying-foxes in East-Asian subtropical island forests depended on the availability of the most abundant bat-figs that serve as predominant foods to bats. Intermediate levels of crop sizes of theses figs appear most fit with their solitary foraging. At sites where density and basal area coverage of these bat-figs were below a certain level, their ability to attract and support foraging fruit bats appeared dwindled, which in a long run may lead to risks to both parties. This calls for considering fruit bat conservation with sufficient effort focusing on natural habitat protection [65, 72], particularly ways to aid for forests, figs, and fig-bat interactions.
